# Precision of the Kalon Herpes Simplex Virus Type 2 IgG ELISA: an international inter-laboratory assessment

**DOI:** 10.1186/s12879-015-1130-6

**Published:** 2015-09-30

**Authors:** Eshan U. Patel, Jordyn Manucci, Erin M. Kahle, Jairam R. Lingappa, Rhoda Ashley Morrow, Estelle Piwowar-Manning, Anelet James, Kwitaka F. Maluzi, Maina M. Cheeba, Glenda Gray, Sinead Delany-Moretlwe, Mubiana Inambao, Bellington Vwalika, Thomas C. Quinn, Oliver Laeyendecker

**Affiliations:** Laboratory of Immunoregulation, Division of Intramural Research, NIAID, NIH, Baltimore, MD USA; Department of Medicine, Johns Hopkins University, Baltimore, MD USA; Department of Epidemiology, University of Washington, Seattle, WA USA; Departments of Medicine, Global Health, and Pediatrics, University of Washington, Seattle, WA USA; Department of Laboratory Medicine, University of Washington, Seattle, WA USA; Vaccine and Infectious Disease Division, Fred Hutchinson Cancer Research Center, Seattle, WA USA; Department of Pathology, Johns Hopkins University School of Medicine, Baltimore, MD USA; Department of Paediatrics and Child Health, Stellenbosch University, Stellenbosch, South Africa; Zambart, Lusaka, Zambia; Perinatal HIV Research Unit, University of the Witwatersrand, Johannesburg, South Africa; Wits Reproductive Health and HIV Institute and University of the Witwatersrand, Johannesburg, South Africa; Zambia-Emory Research Project and Ndola Central Hospital, Ndola, Zambia; Zambia-Emory Research Project, Lusaka, Zambia; NIAID, NIH and SOM, JHU, 855 North Wolfe St., Rangos Building, Room 538A, Baltimore, MD 21205 USA

**Keywords:** Herpes, HSV-2, Kalon, Precision, Reproducibility, Accuracy, Sensitivity, Specificity, Zambia, South Africa

## Abstract

**Background:**

The commercial Kalon HSV-2 IgG ELISA is currently recommended for research use in sub-Saharan Africa because of its superior accuracy compared to other serologic assays. However, there are no data on key precision parameters of Kalon such as inter-operator variation, repeatability, and reproducibility, thus contributing to a barrier for its acceptance and use in clinical trials in sub-Saharan Africa. We evaluated the analytical and field precision of the Kalon HSV-2 IgG ELISA.

**Methods:**

A total of 600 HIV-infected and uninfected serum samples from South Africa and Zambia, previously tested by the gold standard University of Washington HSV western blot (UW-WB), were tested using Kalon by two technologists in an United States reference laboratory. Aliquots of 183 samples were retested using Kalon by an on-site technologist in a South African laboratory and a Zambian laboratory.

**Results:**

Intra-assay variation was below 10 %. Intra-assay, intra-laboratory, and inter-laboratory correlation and agreement were significantly high (*p* < 0.01). In comparison to the UW-WB, accurate performance of Kalon was reproducible by each operator and laboratory. Receiver operating characteristic curve analysis indicated high selectivity of Kalon in the overall study population (area under the curve = 0.95, 95%CI = 0.92–0.97).

**Discussion:**

Kalon is a robust assay with high precision and reproducibility. Accordingly, operator errorlikely does not contribute to the variability observed in Kalon’s specificity throughout sera from sub-Saharan Africa.

**Conclusions:**

In populations with optimal diagnostic accuracy, Kalon is a reliable stand-alone method for on-site HSV-2 IgG antibody detection.

**Electronic supplementary material:**

The online version of this article (doi:10.1186/s12879-015-1130-6) contains supplementary material, which is available to authorized users.

## Background

Herpes simplex virus type 2 (HSV-2) infection is difficult to diagnose during subclinical periods. If a patient does present with symptoms, the infection is initially diagnosed by PCR or viral culture, and is confirmed by detection of IgG antibody for glycoprotein G-2 (gG2) [1]. Serological screening is also recommended in several high-risk populations [1, 2]. Additionally, HSV-2 serostatus is a common biomarker in epidemiological research and clinical trials [3–6]. Thus, serologic tests for gG2 are important for the treatment, prevention and study of HSV-2 infection [1, 7].

A gold standard test is the University of Washington HSV Western blot (UW-WB). However, the UW-WB is expensive and technically difficult for screening and clinical trials [1, 8]. Alternatively, there are commercial enzyme-linked immunosorbent assays (ELISAs). The FDA-cleared Focus HerpeSelect-2 IgG ELISA (“Focus”) has been the popular assay in both clinical and research settings, while the Kalon HSV-2 IgG ELISA (“Kalon”) is not FDA-approved and is only recommended for use in secondary research. Both assays have optimal accuracy in industrialized countries, but their performance on sera from developing countries has varied [9–12].

A recent meta-analysis described better overall accuracy of Kalon than Focus in the same study population in sub-Saharan Africa [9]. For Focus, sensitivity and specificity were 99 and 69 %, respectively, at the manufacturers’ index cut-off (>1.1), and were 91 and 85 %, respectively, at an increased cut-off (>3.5). Kalon’s sensitivity and specificity at the manufacturer’s index cut-off (>1.1) were 95 and 91 %, respectively. For both assays, there is heterogeneity in their specificity between study populations within sub-Saharan Africa, however this heterogeneity is more pronounced for Focus [9, 11–18]. Kalon has also been shown to be more specific than Focus in developing countries beyond sub-Saharan Africa such as Papau New Guinea [19]. Even though Kalon often has higher specificity than Focus, there are situations where the higher sensitivity of Focus is more desirable. For example, Legoff et al. (2008) found that Kalon misses primary infections more frequently than Focus when screening populations presenting with genital ulcerations [1, 20]. Thus, it is recommended to evaluate the accuracy of both assays prior to use in intended populations to select the most appropriate assay and cut-off [9].

Although the accuracy of Kalon has been extensively studied, its precision parameters have not been previously described by the manufacturer or in the literature. This knowledge is essential for any use of Kalon. This is particularly important for its expected expansion from epidemiologic research to clinical trials in sub-Saharan Africa, and in populations where its diagnostic accuracy is superior to that of Focus. Therefore, the primary aim of this study was to evaluate the precision of the Kalon HSV-2 IgG ELISA. We also examine its accuracy compared to UW-WB among Zambian and South African sera.

## Methods

### Ethics statement

The present investigation was conducted according to the principles expressed in the Declaration of Helsinki. The Partners in Prevention HSV/HIV Transmission Study protocol and the procedure for written informed consent was approved by the University of Washington Human Subjects Review Committee and the ethics review committees at other organizations involved in the collaboration (Additional file 1) [21]. All participants provided written informed consent and consented serum samples for long-term storage [21].

### Sample population

Samples came from the Partners in Prevention HSV/HIV Transmission Study. The protocol for the Partners Study has been previously described [6, 21]. In brief, the study enrolled 3408 HSV-2/HIV-1 co-infected individuals and their HIV-uninfected heterosexual partners from seven African countries (Botswana, Kenya, Rwanda, South Africa, Tanzania, Uganda and Zambia) between November 2004 and April 2007. Participants were ≥18 years of age and provided written informed consent. Serum samples were consented for long-term storage in the U.S.A. and were stored at −80 °C.

For the present analysis, samples (*N* = 600) were randomly selected without replacement within the following criteria: (1) de-identified serum from study enrollment in the Partners Study, (2) had UW-WB HSV-2 results readily available, and (3) 300 samples each from South Africa and Zambia. We selected sera from South Africa and Zambia due to availability of sample volume and because the performance of Kalon compared to the UW-WB is unknown in Zambian sera.

### Laboratory testing

All HSV-2 testing by the Kalon HSV-2 IgG ELISA (Kalon Biological Ltd., U.K.) was manually conducted between September and November 2013 using the same kit lot number. Kalon testing was performed per manufacturer protocol unless otherwise stated. All samples (*N* = 600) were first tested at the HIV Prevention Trials Network (HPTN) Laboratory Center at the Johns Hopkins University in Baltimore, MD, U.S.A. The HPTN Laboratory Center conducts retrospective QA/QC testing for HPTN trials, and samples were tested in duplicate (within-run) by an operator with >9 years of laboratory experience (“Technologist-Sr.”). Testing was repeated in duplicate (within-run) by an operator with <1 year of laboratory experience and no previous experience running serological assays (“Technologist-Jr.”). A subset of samples (*n* = 183) were selected based on available volumes, and separate aliquots of the same sample were shipped on dry ice to a field HPTN laboratory site in South Africa (SA) and Zambia (ZM). Samples were re-tested in singlet by a lead on-site operator (“Technologist-SA” and “Technologist-ZM”), and again by Technologist-Sr. at the South African laboratory site.

The manufacturer’s calibrator was run in duplicate on all plate runs in the study. The absorbance of the plate was read by a spectrophotometer. Plate readers at the South African and Zambian laboratory sites had an upper limit of detection of 3.0 optical density (OD) units, while the plate reader at the HPTN Laboratory Center did not have this upper limit. The OD units were used to calculate the manufacturer’s index value cut-offs (<0.9, negative; 0.9–1.1, indeterminate; >1.1 positive) as per the manufacturer’s protocol. The previously known UW-WB HSV-2 result was considered the ‘gold-standard’, and operators were blinded to these data.

### Statistical analyses

Analytical precision was defined as intra-assay (within-run) and inter-assay (between-run) repeatability of results produced by Kalon, as performed by Technologist-Jr. and Technologist-Sr. at the HPTN Laboratory Center. Intermediate precision was determined by evaluating intra-laboratory repeatability of Kalon at the HPTN Laboratory Center and the South African laboratory. Field precision of Kalon was assessed as inter-operator repeatability between all laboratory sites.

Continuous and categorical statistical parameters were used to assess repeatability for each type of precision. Continuous variables, such as the OD units of manufacturer’s calibrator and Kalon index values for serum samples, were analyzed using the coefficient of variation (CV) and Pearson’s correlation coefficient (*r*). Differences in mean and median Kalon index values between operators were determined by the two-sample Wilcoxon rank-sum (Mann–Whitney) test. Since Kalon is intended to be a qualitative assay, the categorical agreement of results (negative, indeterminate, positive) was determined within and between operators and laboratory sites using the Cohen’s Kappa coefficient (κ). A κ-value between 0.80–0.90 and 0.90–1.0 denotes strong and almost perfect agreement, respectively [22].

Field precision of Kalon was further characterized by comparing the inter-operator and inter-laboratory reproducibility of accurate results compared to UW-WB as the ‘gold-standard’. This reproducibility analysis excluded indeterminate samples by the UW-WB (*n* = 3), and was repeated using three scenarios: 1) considered indeterminate samples by Kalon as positive; 2) considered indeterminate samples by Kalon as negative; and 3) removed indeterminate samples by Kalon. Accuracy was characterized by sensitivity and specificity (calculated using standard formulas), and by receiver operator characteristics curve (ROC) analysis. ROC curves illustrate the sensitivity versus 1-specificity (false-positive rate) of an assay, and the area under the curve (AUC) is indicative of diagnostic selectivity. An AUC of 1.0 represents a ‘perfect test’ [23].

To assess the optimal index cutoff (1.1 vs 1.5) of Kalon and allow stratification by characteristics of the study population, accuracy of Kalon compared to the UW-WB was determined for the entire study population using Technologist-Sr.’s results from the HPTN Laboratory Center. This analysis excluded 16 indeterminate samples by the UW-WB and considered 10 indeterminate samples by Kalon as negative. Differences in study population characteristics were determined by Pearson’s *χ*^2^ test and two-sample Wilcoxon rank-sum (Mann–Whitney) test for categorical and continuous variables, respectively. Statistical significance was considered as *P* < 0.05, and 95 % confidence intervals (CI) were calculated from a binomial distribution. Statistical analyses were performed in STATA version 14 (StataCorp, College Station, TX) and R version 3.0.1 (R Foundation for Statistical Computing, Vienna, Austria).

## Results

### Sample characteristics

Of the total 600 samples, 305 were from males, 300 were HIV positive, and the median age was 32 (IQR = 27–39). Two samples had missing gender and age data. The sample population included sera collected from 14 individuals with a medical history of GUD within the past 3 months and 13 individuals with clinical presentation of GUD by physical examination at the time of sample collection (4 samples were positive by both approaches); one sample was missing for GUD status by physical examination and medical history. By UW-WB, 106 samples were seronegative, 478 samples were seropositive, and 16 samples had indeterminate results. Of the 300 HIV positive samples, 99.3 % were HSV-2 seropositive by UW-WB.

### Analytical precision

Intra-assay variation of the manufacturer kit calibrator’s OD units, as performed at the HPTN Laboratory Center, was CV = 5.4 % (CV = 4.2 % for Technologist-Jr. and 6.5 % for Technologist-Sr.; *n* = 13 plate runs per operator). Consistent with these results, the average intra-assay variation in sample index values for both operators was also low (CV = 6.3 %; *n* = 1196). Categorical intra-assay agreement was ‘almost perfect’ for both operators, but was higher for Technologist-Sr. (κ = 0.96) compared to Technologist-Jr. (κ = 0.91; Table 1). Inter-assay variation of the manufacturer kit calibrator’s OD units was CV = 10.2 % (CV = 8.7 % for Technologist-Jr. and CV = 11.8 % for Technologist-Sr. for plate runs performed at the HPTN Laboratory Center (*n* = 13 plate runs per operator). The same operator did not retest serum samples on different plate runs so inter-assay repeatability of Kalon index values could not be assessed.

**Table 1:** Intra-assay (within-run) repeatability of the Kalon HSV-2 IgG ELISA^a^

Operator	N	CV	*r*	Agreement ^b^	κ ^b^
Technologist-Jr.	598	6.7 %	0.99*	96.8 %	0.91*
Technologist-Sr.	598	5.9 %	0.98*	98.7 %	0.96*

*Abbreviations*: *CV* coefficient of variation, *r* Pearson’s correlation coefficient, and *κ*Cohen’s kappa coefficient

*P < 0.01

^a^Data based Kalon as performed at the USA site (HPTN Laboratory Center)

^b^Agreement was based on categorical results interpreted by the manufacturer’s index cut-offs (<0.9, negative; 0.9–1.1, indeterminate; >1.1 positive)

### Intermediate precision

**Fig. 1:**
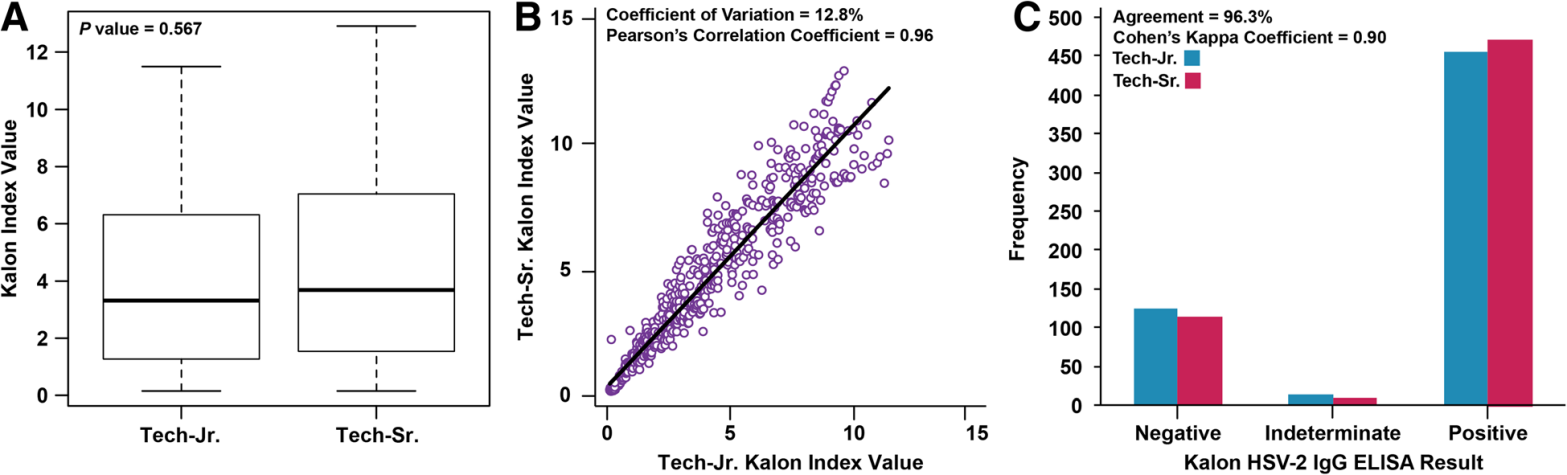
The intermediate precision of the Kalon HSV-2 IgG ELISA (*N* = 596). These data are based on the performance of Kalon by Technologist-Jr. and Technologist-Sr. at the HPTN Laboratory Center in the USA. **a** Shows box plots that represent the range, interquartile range, and median Kalon index values for all samples tested by both operators. **b** Shows the correlation and variability in Kalon index values between operators. **c** Presents the agreement of categorical results between operators as determined by the manufacturer’s index cut-offs (<0.9, negative; 0.9–1.1, indeterminate; >1.1 positive)

Figure 1 depicts the intra-laboratory repeatability of Kalon for all samples tested at the HPTN Laboratory Center. Kalon index values for samples run by Technologist-Jr. (median = 2.48; IQR = 0.42–5.34) and Technologist-Sr. (median = 2.42; IQR = 0.49–5.48) at the HPTN Laboratory Center were not significantly different (*P* = 0.567; Fig. 1a). The sample Kalon index values between both operators had an average CV of 12.8 % and were significantly correlated (*P* < 0.01; Fig. 1b). There was ‘almost perfect’ inter-operator agreement between operators within the HPTN Laboratory Center (κ = 0.90; *n* = 596; Fig. 1c).

In the subset of 183 samples, variation in Kalon index values between operators was twice as high at the South African site (CV = 18.8 %) compared to variation between operators at the HPTN Laboratory Center (CV = 9.6 %). Both correlation (*r* = 0.89) and agreement (κ = 0.89) between operators in the South African laboratory were significantly high, but were lower in comparison to intra-laboratory performance at the HPTN Laboratory Center.

### Field precision

**Fig. 2:**
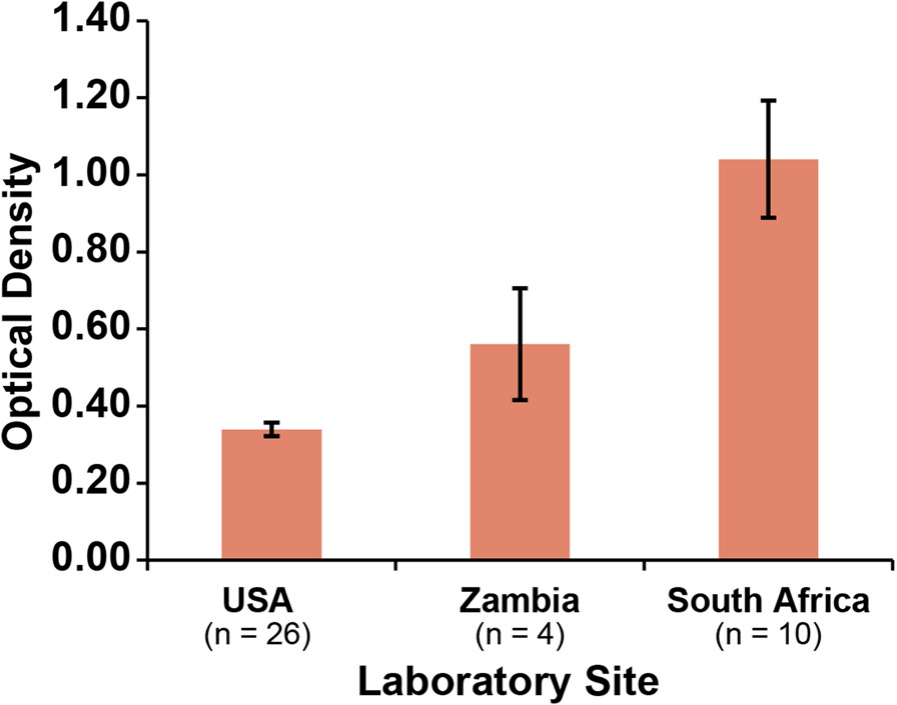
The mean optical density of the Kalon HSV-2 IgG ELISA calibrator for all plate runs in the study

**Fig. 3:**
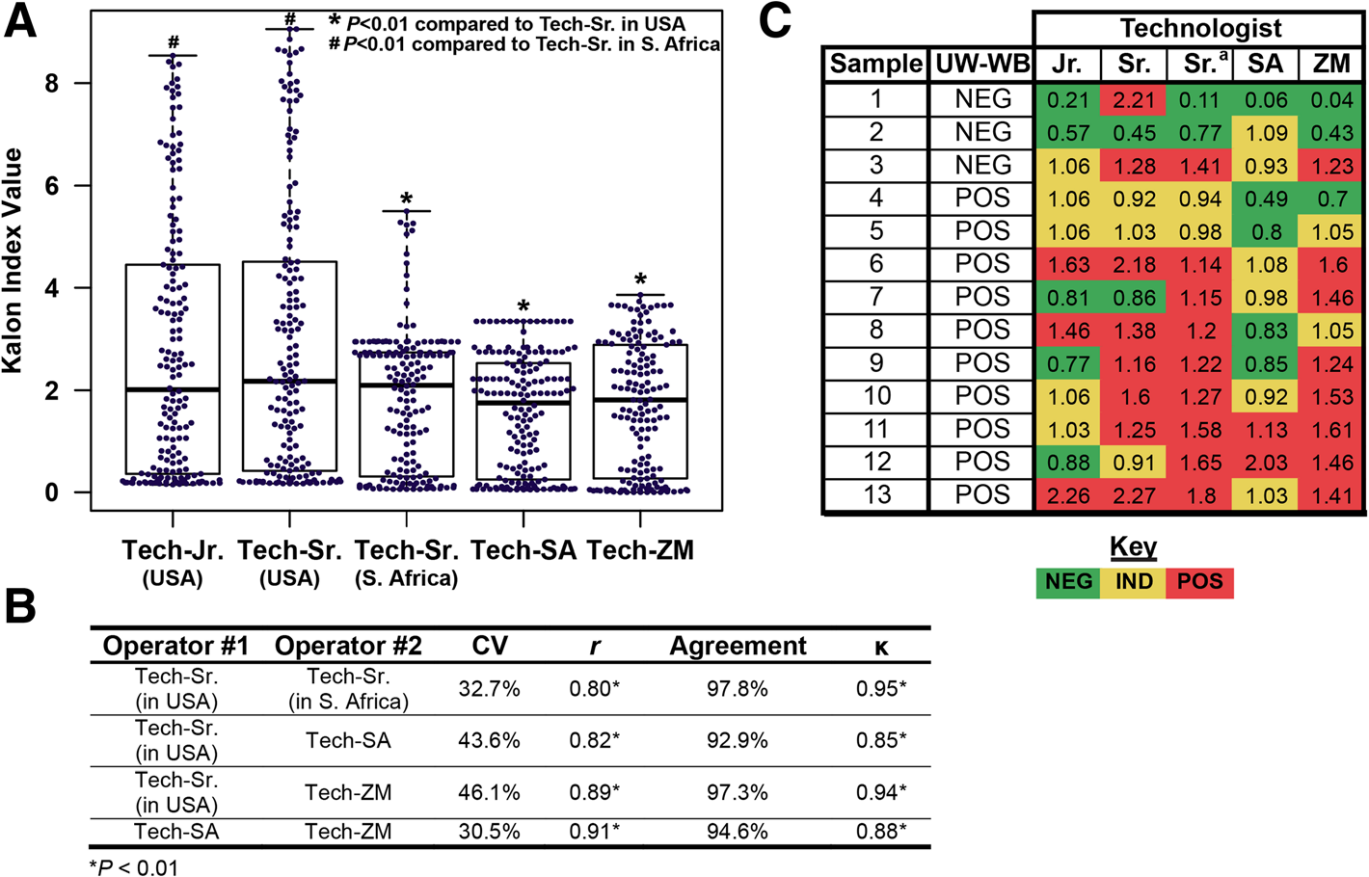
The field precision of the Kalon HSV-2 IgG ELISA. **a** Shows box plots that represent the range, interquartile range, and median Kalon index values for samples tested by all operators (*n* = 170). The *star* and *pound signs* represent inter-operator comparisons by the Wilcoxon rank-sum test. **b** Shows the correlation and variability in Kalon index values between operators and also presents the agreement of categorical results between operators as determined by the manufacturer’s index cut-offs (<0.9, negative; 0.9–1.1, indeterminate; >1.1 positive); (*n* = 170). **c** Depicts any sample that had a Kalon result that was discordant from other Kalon results by all operators (*n* = 183). The ^*a*^ indicates that these results were generated by Technologist-Sr. in South Africa

The mean calibrator OD units were significantly higher in the Zambian and South African laboratory sites compared to performance at the HPTN Laboratory Center (Fig. 2). Of the 183 samples tested by all operators, 13 samples had an OD > 3.0 in the HPTN Laboratory Center and were excluded from continuous comparisons. The mean Kalon index value was significantly higher at the HPTN Laboratory Center (mean index: 2.87) compared to the South African laboratory site (mean index = 1.68) and Zambian laboratory site (mean index = 1.72; *P* < 0.01). The differences in the mean index value between the South African and Zambian laboratories were not significant (*P* = 0.921). Fig. 3a depicts the range of Kalon index values for all operators (*n* = 170). Inter-laboratory variation was higher than intra-laboratory variation, with the CV ranging from 30.5 to 46.1 % (*n* = 170; Fig. 3b).

There was, however, ‘strong’ or ‘almost perfect’ agreement between all operators (*P* < 0.01; *n* = 183; Fig. 3c). Fig. 3c presents the Kalon index values for all samples that were categorically discordant by Kalon between operators. Of the 13/183 samples, 8 samples were considered discordant solely because of indeterminate result(s), as in these samples did not have conflicting results of positive vs. negative between operators. Excluding the 8 indeterminate samples resulted in an overall discordance rate of 2.9 % (5/175) between operators. The majority of samples (10/13) that were discordant between operators were HSV-2 seropositive by UW-WB (Fig. 3c) and none presented with GUD by physical examination or their past medical history (3 months).

In addition to consistency of Kalon results between operators and field sites, the categorical results produced by Kalon and each operator were accurate compared to UW-WB. Performance of Kalon in terms of sensitivity, specificity, and diagnostic selectivity were similar by all operators and field sites (cut-off = 1.1; Table 2). Considering indeterminate samples by Kalon as negative, positive, or excluding them from this analysis had no significant effect on the statistical parameters (Table 2).

**Table 2:** Accurate reproducibility of the Kalon HSV-2 IgG ELISA (*N* = 183; HSV-2 prevalence = 72 %) ^a^

Kalon conditions	Sensitivity, % (95 % CI)	Specificity, % (95 % CI)	AUC (95 % CI)
IND as NEG ^b^			
Technologist-Jr.	90.1 (83.6–94.6)	100.0 (92.7–100.0)	0.95 (0.93–0.98)
Technologist-Sr. ^e^	92.4 (86.4–96.3)	95.9 (86.0–99.5)	0.94 (0.91–0.98)
Technologist-Sr. ^f^	94.7 (89.3–97.8)	98.0 (89.1–99.9)	0.96 (0.94–0.99)
Technologist-SA	88.5 (81.8–93.4)	100.0 (92.7–100.0)	0.94 (0.92–0.97)
Technologist-ZM	93.1 (87.4–96.8)	98.0 (89.1–99.9)	0.96 (0.93–0.98)
IND as POS ^c^			
Technologist-Jr.	93.1 (87.4–96.8)	98.0 (89.1–99.9)	0.96 (0.93–0.99)
Technologist-Sr. ^e^	94.7 (89.3–97.8)	95.9 (86.0–99.5)	0.95 (0.91–0.99)
Technologist-Sr. ^f^	96.2 (91.3–98.7)	98.0 (89.1–99.9)	0.97 (0.95–1.00)
Technologist-SA	91.6 (85.5–95.7)	95.9 (86.0–99.5)	0.94 (0.90–0.97)
Technologist-ZM	94.7 (89.3–97.8)	98.0 (89.1–99.9)	0.96 (0.94–0.99)
IND excluded ^d^			
Technologist-Jr.	92.9 (87.0–96.7)	100.0 (92.6–100.0)	0.97 (0.94–0.99)
Technologist-Sr. ^e^	94.5 (89.1–97.8)	95.9 (86.0–99.5)	0.95 (0.92–0.99)
Technologist-Sr. ^f^	96.1 (91.2–98.7)	98.0 (89.1–99.1)	0.97 (0.94–1.00)
Technologist-SA	91.3 (85.0–95.6)	100.0 (92.5–100.0)	0.96 (0.93–0.98)
Technologist-ZM	94.6 (89.1–97.8)	98.0 (89.1–99.9)	0.96 (0.94–0.99)

*Abbreviations*: *POS* positive, *NEG* negative, *IND* indeterminate, *CI* confidence interval, *AUC* area under the receiver operating characteristic curve

^a^Gold standard: UW-WB; indeterminate results by UW-WB were excluded in the analysis (*n* = 3)

^b^Indeterminate results by Kalon were considered as negative in the analysis

^c^Indeterminate results by Kalon were considered as positive in the analysis

^d^Indeterminate results by Kalon were excluded in the analysis

^e^Kalon was performed at the HPTN Laboratory Center (USA site)

^f^Kalon was performed in the South African laboratory

### Diagnostic accuracy

In the overall study population, the optimal cut-off was 1.1 (AUC = 0.95, 95 % CI = 0.92, 0.97) when excluding 16 indeterminate UW-WB samples and considering 10 indeterminate Kalon results as negative (Table 3). Country of origin did not significantly affect the diagnostic accuracy as defined by the AUC, however, specificity was lower in Zambian sera (88.7, 95 % CI = 77.0, 95.7) than in South African sera (98.1, 95 % CI = 89.9, 100.0; Table 3). Of note, sera from Zambia were more likely to be from older (*P* = 0.021) and HIV positive (*P* = 0.012) individuals compared to sera from South Africa. Although there was a slightly higher prevalence of GUD in sera from Zambia compared to South Africa, the difference was not significant (Table 3). Additionally, all GUD positive samples by physical examination and medical history were concordantly seropositive by UW-WB and Kalon. Raising the cut-off to 1.5 improved specificity in Zambian sera, but had no significant effect on diagnostic selectivity since it also decreased the assay’s sensitivity from 97.0 % (cut-off = 1.1) to 92.3 % (cut-off = 1.5) (Table 3).

**Table 3:** Diagnostic accuracy of the Kalon HSV-2 IgG ELISA compared to UW-WB in South African and Zambian sera (*N* = 600) ^a^

Characteristic	Country of origin		HIV status		Total (*n*/*N*)
	South Africa	Zambia	HIV negative	HIV positive	
HSV-2 Prevalence by UW-WB, % (95 % CI)	82.1 (77.2, 86.3)	81.6 (76.6, 85.9)	63.9 (58.0, 69.4)	99.3 (97.6, 99.9)	81.9 (478/584)
GUD Presentation by Physical Exam, % (95 % CI)	1.4 (0.4, 3.4)	3.5 (1.7, 6.3)	0.7 (0.1, 0.2)	4.1 (2.1, 7.0)	2.4 (14/583)
Medical History of GUD in past 3 months, % (95 % CI)	1.7 (0.6, 3.9)	3.1 (1.4, 5.8)	0.7 (0.1, 0.2)	4.1 (2.1, 7.0)	2.4 (14/583)
HIV Prevalence, % (95 % CI)	49.7 (43.8, 55.5)	51.7 (45.8, 57.6)	–	–	50.7 (296/584)
Median Age (IQR)	31.6 (27.1, 37.4)	32.2 (27.2, 41.7)	33.4 (28.0, 39.6)	31.1 (26.3, 37.6)	32.0 (27.1, 38.7)
Kalon Performance (cut-off = 1.1)					
Sensitivity, % (95 % CI)	94.2 (90.5, 96.8)	97.0 (94.0, 98.8)	94.0 (89.6, 97.0)	96.6 (93.8, 98.4)	95.6 (93.4, 97.3)
Specificity, % (95 % CI)	98.1 (89.9, 100.0)	88.7 (77.0, 95.7)	94.2 (87.9, 97.9)	–	93.4 (86.9, 97.3)
AUC (95 % CI)	0.96 (0.94, 0.99)	0.93 (0.88, 0.97)	0.94 (0.91, 0.97)	–	0.95 (0.92, 0.97)
Kalon Performance (cut-off = 1.5)					
Sensitivity, % (95 % CI)	92.2 (88.1, 95.2)	92.3 (88.2, 95.4)	88.6 (83.1, 92.8)	94.6 (91.3, 96.9)	92.3 (89.5, 94.5)
Specificity, % (95 % CI)	98.1 (89.9, 100.0)	92.5 (81.8, 97.9)	96.2 (90.4, 98.9)	–	95.3 (89.3, 98.5)
AUC (95 % CI)	0.95 (0.93, 0.98)	0.92 (0.88, 0.96)	0.92 (0.89, 0.95)	–	0.94 (0.91, 0.96)

*Abbreviations*: UW-WB, University of Washington HSV Western Blot; CI, confidence interval; GUD, Genital Ulcer Disease, AUC, area under the curve

^a^Indeterminate results by UW-WB were excluded in the analysis (*n* = 16). Kalon data are based on its performance by Technologist-Sr. at the HPTN Laboratory Center, and indeterminate results by Kalon (*n* = 10) were considered seronegative

Due to the high seroprevalence of HSV-2 (99.3 %) among the HIV positive samples, specificity and the AUC for this population could not be assessed. Characteristics of the indeterminate samples by UW-WB and Kalon are presented in Table 4. Of the 16 indeterminate samples by UW-WB, 10 (62.5 %) were positive by Kalon. No indeterminate samples by UW-WB or Kalon had symptoms of GUD in their medical history (past 3 months) or had physical presentation of GUD (Table 4).

**Table 4:** Characteristics of the indeterminate samples by UW-WB and Kalon (index cut-off = 1.1)

	Indeterminate by UW-WB	Indeterminate by Kalon (index: 0.9–1.1) ^a^
Total (No.)	16	10
GUD Presentation by Physical Exam (No.)	0	0
Medical History of GUD in past 3 months (No.)	0	0
HIV Prevalence, % (n/N)	25.0 (4/16)	30.0 (3/10)
Median Age, years (IQR)	33.0 (25.9, 39.2)	31.4 (26.4, 35.8)
Country of Origin (No.)		
South Africa	4	5
Zambia	12	5
Kalon Result (No.) ^a^		
Negative	5	–
Indeterminate	1	–
Positive	10	–
UW-WB (No.)		
Negative	–	2
Indeterminate	–	1
Positive	–	7

*Abbreviations*: *UW*-*WB* University of Washington HSV Western Blot, *GUD* genital ulcer disease, and *IQR* interquartile range

^a^Kalon results were produced in the HPTN Laboratory Center (USA site) by Technologist-Sr. and were interpreted by the manufacturer’s index cut-offs: (<0.9, negative; 0.9–1.1, indeterminate; >1.1, positive)

## Discussion

It is estimated that 19.2 million individuals were newly infected with HSV-2 infection in 2012. Given the global estimate of HSV-2 prevalence of 11.3 %, with significant burden in sub-Saharan Africa (32 %) [24], it is essential to keep clinicians and researchers informed of all characteristics of HSV diagnostics. Unlike FDA-approved, commercially available, serologic HSV-2 assays, the Kalon HSV-2 IgG ELISA has not been rigorously assessed beyond diagnostic accuracy. This study demonstrates that Kalon has a high level of analytical precision. Despite inter-laboratory variation in its optical density and index values, this qualitative ELISA was able to consistently categorize HSV-2 serostatus within and between a quality assurance site and field laboratories. Optimal reproducibility of Kalon was maintained across operators with varying levels of experience running serological assays. Taken together, in study populations where its accuracy compared to UW-WB is optimal, Kalon should be considered a reliable test for HSV-2 serodiagnostics.

Resource-limited settings are heavily burdened by HSV-2 infection. Although Kalon has been shown to have optimal accuracy in several populations, its utility in field research laboratories has not been widely accepted. The optimal repeatability of Kalon observed in this analysis suggests that Kalon can be performed in resource-poor regions as a stand-alone method for HSV-2 serology. This is especially important for large-scale HIV/HSV-2 epidemiological investigations such as the HPTN 071 PopART community randomized trial in South Africa and Zambia [25, 26]. Rather than shipping all samples to laboratories in developed countries solely for HSV-2 screening, use of Kalon by on-site operators in field laboratories is a more feasible and cost-effective alternative.

This study confirms that Kalon can perform accurately compared to UW-WB. Specificity of Kalon, compared to the UW-WB, was previously unknown in Zambia, however, our finding of 98.1 % specificity in South Africa is higher but comparable to a study that found 85 % (95 % CI, 61–100 %) specificity (index cut-off = 1.1) [18]. Specificity was slightly lower in Zambian sera (88.7 %; index cut-off = 1.1) compared to in South African sera in this study population, and it was previously noted that operator error might explain the variability of Kalon’s specificity throughout sub-Saharan Africa [8, 9]. However, our study reveals that differential laboratory performance of Kalon is likely not a major contributor to regional differences in its accuracy compared to UW-WB.

The regional variability in Kalon’s specificity observed in this study may be in part due to differences in study population characteristics. Although this study was not powered to assess the effect of HIV on Kalon’s specificity, previous studies have reported reduced specificity among HIV-infected individuals and our study had a slightly higher HIV prevalence in the Zambian vs. South African study population [9, 18]. As hypothesized by previous serodiagnostic validation studies, lower specificity of Kalon compared to UW-WB may also be due to cross-reactivity with unidentified antibodies or a nucleotide polymorphism in the gG2 sequence among African populations [8, 9, 12, 14]. Recent studies support the latter hypothesis as we now know there is regional nucleotide and antigenic variation in the HSV glycoprotein—the diagnostic target of HSV serologic assays [27, 28]. Until improved serologic assays are available, it remains necessary to evaluate Kalon’s accuracy in proposed populations prior to their utility [9].

This study had several limitations. In terms of the analytic precision analysis, we did not evaluate differential lot performance. Although we report inter-laboratory variability in the OD and Kalon index values, we did not identify operational (equipment) and environmental factors (e.g. temperature, pH of water, and humidity) that may have contributed to inter-laboratory variability. Further work is needed to pinpoint why the OD values were higher at the sub-Saharan African sites compared to the United States. In addition, due to the high HSV-2 seroprevalence in this study population, the applicability of our accuracy analysis may only apply to high prevalence settings where seroconversion is a common event.

A limitation of serological assays in general is that they may miss any persons undergoing seroconversion. One study demonstrated that UW-WB and Kalon have a median time to seroconversion of 87 and 120 days, respectively, thereby missing recently infected individuals [29]. We conservatively considered indeterminate results by Kalon as negative in the accuracy analysis, but indeterminate results may be an indication of early seroconversion [30]. Most indeterminate results by Kalon were seropositive by UW-WB, and the majority of discordant Kalon results between operators were also seropositive by UW-WB. This suggests that Kalon may lack sensitivity compared to UW-WB. Interestingly, most indeterminate samples by UW-WB were seropositive by Kalon. Although indeterminate samples did not significantly affect reproducibility, a higher prevalence of indeterminate samples in a study population may prove to be problematic for other populations. The root cause for indeterminate samples, whether early seroconversion, genetic variation of the infecting virus or cross-reactivity due to different infections, warrants further investigation.

Meanwhile, indeterminate samples are challenging for clinicians and researchers. In a clinical setting, indeterminate samples by Focus (currently used as a stand-alone method) require follow-up testing at a later date to demonstrate seroconversion. Alternatively, confirmation testing by western blot is often conducted in clinical trials where follow-up sera may not be available [21]. It may be plausible to adapt the same testing algorithms for Kalon. In secondary research studies, the protocol for handling indeterminate samples will likely be dependent on the study outcome. Further work is needed to develop and optimize serologic HSV-2 assays, as serology remains the test of choice for HSV-2 screening.

## Conclusions

In summary, the Kalon HSV-2 IgG ELISA provides reliable results for determining HSV-2 serostatus. While it is preferable to use FDA-cleared assays for diagnostics, these data confirm that Kalon is a dependable replacement for use in populations where its accuracy is superior to current methods. These data also suggest that Kalon can be utilized in field laboratories of resource-limited settings, enhancing the feasibility to monitor the epidemic and assess intervention efforts. Particularly in sub-Saharan Africa where HSV-2 diagnostics are challenging, it may be of benefit to extend Kalon’s utility beyond epidemiological research.

## Additional file

Additional file 1:
**Ethics review committees from organizations that approved the study protocol for the Partners in Prevention HSV/HIV Transmission Study.** (DOCX 13 kb)

## Abbreviations

HSV-2: Herpes Simplex Virus Type 2
gG2: Glycoprotein-G2
UW-WB: University of Washington Western blot
ELISA: Enzyme-linked immunosorbent assay
Focus: Focus HerpeSelect-2 IgG ELISA
Kalon: Kalon HSV-2 IgG ELISA
FDA: United States Food and Drug Administration
HIV: Human immunodeficiency virus
HPTN: HIV Prevention Trials Network
SA: South Africa
ZM: Zambia
CV: Coefficient of variation
R: Pearson’s correlation coefficient
Κ: Cohen’s Kappa coefficient
ROC: Receiver operating characteristic
AUC: Area under the curve
CI: Confidence interval
