# Erratum to: Precision of the Kalon Herpes Simplex Virus Type 2 IgG ELISA: an international inter-laboratory assessment

**DOI:** 10.1186/s12879-017-2454-1

**Published:** 2017-05-19

**Authors:** Eshan U. Patel, Jordyn Manucci, Erin M. Kahle, Jairam R. Lingappa, Rhoda Ashley Morrow, Estelle Piwowar-Manning, Anelet James, Kwitaka F. Maluzi, Maina M. Cheeba, Glenda Gray, Barry Kosloff, Sinead Delany-Moretlwe, Mubiana Inambao, Bellington Vwalika, Thomas C. Quinn, Oliver Laeyendecker

**Affiliations:** 1 Laboratory of Immunoregulation, Division of Intramural Research, NIAID, NIH, Baltimore, MD USA; 2 Department of Medicine, Johns Hopkins University, Baltimore, MD USA; 3 Department of Epidemiology, University of Washington, Seattle, WA USA; 4 Departments of Medicine, Global Health, and Pediatrics, University of Washington, Seattle, WA USA; 5 Department of Laboratory Medicine, University of Washington, Seattle, WA USA; 6 Vaccine and Infectious Disease Division, Fred Hutchinson Cancer Research Center, Seattle, WA USA; 7 Department of Pathology, Johns Hopkins University School of Medicine, Baltimore, MD USA; 8 Department of Paediatrics and Child Health, Stellenbosch University, Stellenbosch, South Africa; 9 Zambart, Lusaka, Zambia; 10 Perinatal HIV Research Unit, University of the Witwatersrand, Johannesburg, South Africa; 11 Wits Reproductive Health and HIV Institute and University of the Witwatersrand, Johannesburg, South Africa; 12 Zambia-Emory Research Project and Ndola Central Hospital, Ndola, Zambia; 13 Zambia-Emory Research Project, Lusaka, Zambia; 14 NIAID, NIH and SOM, JHU, 855 North Wolfe St., Rangos Building, Room 538A, Baltimore, MD 21205 USA; 15 London School of Hygiene and Tropical Medicine, London, UK; 16 ZAMBART, UNZA School of Medicine, Lusaka, Zambia

## Erratum

After the publication of this work [1] it was noticed that the 11^th^ author, Barry Kosloff, was omitted from the author list.

The affiliation for Barry Kosloff was also omitted and should be included in the Author details section as follows:

London School of Hygiene and Tropical Medicine, London, UK; ZAMBART, UNZA School of Medicine, Lusaka, Zambia.

The Authors’ contributions section should also include the sentence below:

Barry Kosloff (BK) supervised Kalon HSV2 testing in the ZAMBART Laboratory performed for this publication.

The author list and affiliation have been corrected accordingly in this erratum.
