# Supplementary material for: Precision of the Kalon Herpes Simplex Virus Type 2 IgG ELISA: an international inter-laboratory assessment
Source: BMC Infect Dis. 2015 Sep 30;15:398. doi: 10.1186/s12879-015-1130-6 (PMC4591065; doi:10.1186/s12879-015-1130-6)
Supplement: Additional file 1: — Ethics review committees from organizations that approved the study protocol for the Partners in Prevention HSV/HIV Transmission Study. (DOCX 13 kb) [file 12879_2015_1130_MOESM1_ESM.docx]

**ADDITIONAL FILE #1**

**Ethical Approval**

In the Partners in Prevention HSV/HIV Transmission Study, ethics committees at the following institutions approved the study protocol:

South African sites:

- University of Witwatersrand
- University of Cape Town

Zambian sites:

- Tropical Disease Research Centre, Republic of Zambia National Ethics Committee
- Emory University

Botswana site:

- Republic of Botswana Ministry of Health
- Harvard School of Public Health

Rwandan site:

- Republic of Rwanda National Ethics Committee, Emory University

Tanzanian site:

- Kilimanjaro Christian Medical College
- Harvard School of Public Health
- London School of Hygiene and Tropical Medicine

Ugandan site:

- Uganda National Ethics Committee

Kenyan sites:

- Kenyatta National Hospital
- Moi University
- Kenya Medical Research Institute
- Indiana University
- University of California San Francisco
- University of Washington
